# Goal Missed, Self Hit: Goal-Setting, Goal-Failure, and Their Affective, Motivational, and Behavioral Consequences

**DOI:** 10.3389/fpsyg.2021.704790

**Published:** 2021-09-21

**Authors:** Jessica Höpfner, Nina Keith

**Affiliations:** Department of Psychology, Technical University of Darmstadt, Darmstadt, Germany

**Keywords:** goal-setting theory, goal-failure, affect, self-esteem, motivation, task choice

## Abstract

Setting high and specific goals is one of the best-established management tools to increase performance and motivation. However, in recent years, potential downsides of goal-setting are being discussed. One possible downside is the high risk of failing the goal. In an approach to integrate research on the consequences of goal-failure and the basic assumptions of goal-setting theory, we investigated whether failure of a high and specific goal has detrimental effects on a person’s affect, self-esteem, and motivation. In Experiment 1, 185 participants received fictitious feedback about attaining or failing an assigned high and specific goal. In Experiment 2 with 86 participants, we manipulated goal-failure through task-difficulty and we included task choice as a behavioral measure of motivation. In both experiments, participants who failed the high and specific goal showed a decrease in affect, self-esteem, and motivation compared to participants who attained that goal. Results indicate that failing a high and specific goal can be damaging for self-related factors that may be crucial for organizational long-term outcomes. We advise organizations to consider potential undesirable effects when using goal-setting interventions.

## Introduction

Over 1,000 studies have consistently shown that setting high and specific goals is linked to increased task performance, persistence, and motivation, compared to vague or easy goals (Locke and Latham, 2002, 2006). Given this empirical evidence, setting high (which means a high difficulty that only a certain percentage of individuals can reach) and specific (which means tangible information on what needs to be attained) goals has become a highly recommended motivational and leadership tool in organizations. However, in recent years, more and more studies raised concerns about possible undesirable effects of goal-setting. For example, goals can narrow the attention focus on goal-related actions, so that other important issues are missed (Ordóñez et al., 2009), goals may increase risk-taking and unethical behavior (Neale and Bazerman, 1985; Knight et al., 2001; Schweitzer et al., 2004), inhibit learning (Earley et al., 1989; Cervone et al., 1991), or create an overly competitive environment (Mitchell and Silver, 1990).

The current research seeks to shed light on another possible downside of setting high and specific goals: the possibility of goal-failure and the associated negative consequences. Locke and Latham (1990, p. 349) advocated that (at least in laboratory settings) a high and specific goal “that only 10% of the subjects can reach” should be set to achieve maximum individual performance (see, e.g., Locke et al., 1989; Latham and Locke, 1991; Latham and Seijts, 1999; Welsh and Ordóñez, 2014; Welsh et al., 2019). However, this implies that only 10% of individuals are able to attain the high and specific goal and 90% will fail the goal. What happens to those who fail the high and specific goal? Several theories have discussed possible processes induced by goal-failure in general (Dweck and Leggett, 1988; Carver and Scheier, 1990; Judge and Kammeyer-Mueller, 2004), but there is a dearth of empirical research on the consequences of failure of a high and specific goal.

We argue that failing a high and specific goal induces several processes that can harm a person’s affect, self-esteem, and motivation. Reducing such self-related factors can have serious consequences for the person as well as the organizations, for example reduced extrarole performance (Judge and Kammeyer-Mueller, 2008), reduced organizational citizenship behavior (Welsh et al., 2020), or increased absenteeism (Shi et al., 2013). Decreased motivation may also lead to disengagement from challenging tasks (Seo and Ilies, 2009) or choosing tasks with low difficulty (Nichols et al., 1991).

While there is some evidence on the effects of goal-failure on affect (e.g., Martin et al., 1993; Grieve et al., 1994), to our knowledge there are little to no studies that integrate research of failure with the basic assumptions of goal-setting theory. Hence, the present research seeks to close this research gap, first, by replicating known effects of goal-failure on affect while using a high and specific goal and, second, by investigating the effects on additional self-related factors such as self-esteem and motivation that are also crucial for organizational outcomes. In the next sections, we will outline the underlying theories and potential processes that may lead to negative consequences after goal-failure of a high and specific goal. We will describe in detail the expected effects of goal-failure for affect, self-esteem, and motivation. We will then describe two experimental studies we conducted to examine those effects.

## Theory and Hypotheses

Setting high and specific goals is the basic recommendation by goal-setting theory to increase performance (Locke and Latham, 1990, 2002, 2006); however, failing these goals may induce processes that are damaging for one’s self. Goals can be described as objects of a person’s ambition that direct attention to goal-relevant activities, mobilize effort, and motivate to develop task-relevant strategies for goal-attainment (Locke et al., 1981). In over 35years of research, Locke and Latham (2002) developed goal-setting theory to influence, predict, and explain performance on organizational tasks through goals. Their core findings were that high and specific goals increased performance, persistence, and motivation compared to vague or so-called “do-your-best” goals (Locke and Latham, 1990).

However, most past research focused on these core findings and increasing performance as the main outcome, while ignoring potential detrimental effects on intrapersonal and self-related factors, especially when the high and specific goal is failed. Some evidence was found that high and specific goals lead to a decrease in affect, because individuals evaluate their performance relatively to a reference point (Oliver et al., 1994; Thompson, 1995; Galinsky et al., 2002). Even individuals who had objectively good outcomes felt worse when they had a high and specific goal as their reference point (Thompson, 1995; Galinsky et al., 2002). What happens when individuals fall *under* their reference point? Surprisingly, there is a lack of research on the consequences of failing a high and specific goal. It is important to examine the consequences of goal-failure of a high and specific goal since they are the key element of goal-setting interventions in organizations. We propose that failing the high and specific goal may induce detrimental processes for several intrapersonal and self-related factors. We chose intrapersonal factors that have been consistently demonstrated to be strongly connected with organizational outcomes and hence impairing those has the potential to harm the employee and the organization in the long-run.

First, we propose that goal-failure of a high and specific goal can damage a person’s affect. A person’s affect, which is a common indicator for well-being (Sonnentag, 2015), refers to the positive or negative personal reactions to experiences (Lazarus, 1982). Affect is often used as an umbrella term for mood, emotions, and evaluations. One can experience pleasant emotions or unpleasant ones (Diener, 2000). Several theories support the notion that goal-failure can be harmful for a person’s affect. First, self-regulation theory suggests that behavior is meta-monitored by the individual and people seek to reduce discrepancy between their present actions and a reference value. If their progress toward that reference value is sub-standard, they experience negative affect (Carver and Scheier, 1990; Moberly and Watkins, 2010). Second, achievement goal theory suggests that individuals with a focus on an externally-set standard view their skillset as fixed and unchangeable (Dweck and Leggett, 1988). Failing the standard for them then implies that their skills are insufficient and they view the failure as a negative judgement of their competence. Thus, when individuals fail a high and specific goal, they experience a discrepancy between their skills and the goal and will experience negative affect. Negative affect can lead to severe consequences like reduced performance (Seo and Ilies, 2009), exhaustion (Halbesleben and Wheeler, 2011), counterproductive work behavior (Scott and Barnes, 2011), and in the long-run even to burnout, which is related to increased absenteeism (Ybema et al., 2010).

A number of studies have examined the consequences of goal-failure for a person’s affect. However, these studies do not directly relate to goal-setting theory. For example, in one experiment, goal level (primary vs. subgoals) and feedback of success or failure were manipulated. Participants who received a primary goal and feedback of goal-failure showed highest negative affect and decreased expectancy for future performance (Houser-Marko and Sheldon, 2008). In another study, participants reported their negative affect; their ruminative self-focus, as well as their current goal and the importance of that goal eight times daily over 7 days (Moberly and Watkins, 2010). It was found that low goal-success and high goal-importance were associated with high negative affect. Rumination after experiences of failure was also examined in another investigation, in which failure to attain prevention or promotion goals was manipulated by letting participants recall past failure experiences (Jones et al., 2013). It was found that failure experiences lead to increased rumination and intensified negative affect, especially for promotion goal failures. In a summary on goals and affect, Plemmons and Weiss (2013) gathered previous findings on the effects of goal-failure on subsequent affect. They concluded that goal-attainment has positive effects on affect, whereas goal-failure has negative effects on affect (see Plemmons and Weiss, 2013, pp. 121). None of these studies involved high and specific goals. We found two exceptions where high goals according to goal-setting theory were used. In one study, goal-success and goal-failure were used as mood-inducing method (Henkel and Hinsz, 2004). In another investigation, goal-difficulty, goal source, and failure tolerance were manipulated in a scenario experiment in which participants were confronted with a character who fails his fictitious exam (Kim and Clifford, 1988). It was found that for very difficult goals that were assigned by someone else, feelings after failure tended to be more negative. However, the authors did not find unambiguous support for the relationship between goal-difficulty and responses to goal-failure, and the goal only was presented as an item on the scenario booklet; participants did not have to complete the goal themselves.

Hence, there is some empirical evidence that goal-failure may have detrimental effects for an individual’s affect; however, research is needed to test this effect for high and specific goals that are the basic recommendation of goal-setting theory. We propose that:

*H1*: Individuals who fail their high and specific goal will show a more negative affect than individuals who attain their high and specific goal.

Second, we propose that goal-failure of a high and specific goal can damage a person’s self-esteem. A person’s self-esteem reflects their evaluation of themselves and their abilities (Rosenberg, 1965). Identity theory describes self-esteem as an outcome of the ratio between successes and goals (Stets and Burke, 2000; Stryker and Burke, 2000), meaning the degree to which individuals are able to match their identity goal with their actual performance. If their identity goal matches with their actual performance, self-verification is successful. Successful self-verification leads to higher self-esteem. In contrast, disruption of the self-verification process, for example goal-failure, can have negative consequences for a person’s self-esteem. Reduced self-esteem can have severe long-term consequences, for the individual as well as for the organization, for example increased turnover cognitions/intentions (Gardner and Pierce, 2001), decreased citizenship behavior (Lee, 2003), and lower organizational commitment (Van Dyne and Pierce, 2004). Hence, it is crucial to examine the consequences of goal-failure for self-esteem.

There is only a small body of research on the consequences of failure for a person’s self-esteem. In one study, it was found that participants who received poor exam scores showed reduced self-esteem (Heatherton and Polivy, 1991). The same was found when failure was manipulated by assigning a puzzle task that was impossible to solve in the given time. Participants in the failure condition showed reduced self-esteem after the task. However, in both studies, there was no assigned high and specific goal. Both studies examined perceived failure on self-esteem and did not measure whether participants had a goal prior to the exam or the task. In another series of experiments, achievement goals were unconsciously activated with several methods (Bongers et al., 2010). Participants then performed different tasks that were either easy or difficult to solve. Participants primed with achievement goals reported lower levels of self-esteem after the difficult tasks throughout all experiments. However, there were no assigned high and specific goals and success and failure were not manipulated, but depended on task difficulty condition, meaning that all participants in the difficult task condition were classified as having failed the goal, even though the goal to achieve was only unconscious and neither high nor specific.

Considering these previous findings, it becomes obvious that there are some indications that failure and more specifically goal-failure may have detrimental effects for a person’s self-esteem. The present research seeks to examine these effects when using high and specific goals. We propose that:

*H2*: Individuals who fail their high and specific goal show lower self-esteem than individuals who attain their high and specific goal.

Third, we propose that goal-failure of a high and specific goal can reduce motivation for future tasks. Work-related motivation is one of the most common topics in organizational psychology and is described as “an umbrella term meant to capture the dense network of concepts and their interrelations that underlie observable changes in the initiations, direction, intensity, and persistence of voluntary action” (Kanfer et al., 2017, p. 339). Hence, we base our conceptualization of work motivation on the voluntarily change of intensity and persistence of an action toward any work-related activity. Work motivation affects how individuals develop their skills, the careers that they pursue, how they allocate their resources, and also affects how activities during work are tackled (Kanfer et al., 2017). Setting high and specific goals is one of the best-known methods to increase work motivation. If the goal is failed, however, we propose that several other processes can be activated that are detrimental to motivation.

According to achievement goal theory, individuals with performance goals avoid challenges when confronted with obstacles, independently of their initial ability (Dweck and Leggett, 1988). While trying to attain a performance goal, individuals feel that their abilities are measured. When goal-failure occurs, individuals perceive their abilities as inadequate and themselves as incompetent (Dweck and Leggett, 1988). Individuals who view themselves as competent will react more positively to responsibilities than individuals who see themselves as incompetent (Judge et al., 1997). Accordingly, individuals who perceive themselves as incompetent will react negatively to responsibilities and view themselves as less likely to succeed (Judge et al., 1998). These individuals will react to failure with withdrawal of effort and reduced persistence (Judge and Kammeyer-Mueller, 2004; Yeo and Neal, 2004). Considering the described definition of motivation as changes of intensity and persistence of voluntary actions, we conclude that goal-failure has the potential to reduce a person’s subsequent motivation.

There are few studies which have investigated the effects of goal-failure on subsequent motivation. Two studies manipulated goal type (learning vs. performance goals) and then used fictitious feedback of goal-failure to investigate the effects on subsequent motivation. In one study, students with a performance goal who received feedback of goal-failure performed worse in a subsequent task (Cianci et al., 2010). In this study, subsequent performance was used as an indicator for changes in motivation. In another research, subjects with a performance goal avoided more difficult subsequent tasks after goal-failure (Nichols et al., 1991). In this investigation, subsequent task choice after failure was used as an indicator of changes in motivation. In one study, participants completed a cycling task and received manipulated performance feedback about attaining or failing their assigned goal before completing a subsequent cognitive task (Healy et al., 2015). There were no differences in subsequent performance between goal-failure and goal-attainment conditions. The authors concluded that a physical task may not have been suitable to manipulate goal-failure and that a physical task may enhance cognitive functioning, which could mask the detrimental effects of goal-failure. Again, these studies did not integrate high and specific goals. There is one exception, in which participants actually received a high goal prior to the task (Vohs et al., 2013), but in this experiment, the goal was set so high that it was actually unattainable and thus, again, did not match the basic assumptions of goal-setting theory. It was found that after goal-failure, expectancy for future performance and interest in performing similar tasks, which were used as indicators of motivation, were lower.

Taken together, there is some evidence that goal-failure of performance goals can have undesirable effects on subsequent motivation. We seek to examine these effects when using high and specific goals that are the key element of goal-setting interventions. We propose that:

*H3*: Individuals who fail their high and specific goal show lower motivation than individuals who attain their high and specific goal.

## Overview of Studies

We conducted two experiments in which we manipulated goal-failure to examine the consequences of failure of a high and specific goal. To manipulate goal-failure, we used fictitious feedback in Study 1 and varied task difficulty in Study 2, so that goal-failure is independent from a person’s skill-level. In Study 1, we focused on the person’s affect, self-esteem, and subsequent self-reported motivation after receiving feedback of goal-failure compared to feedback of goal-attainment or no feedback. Study 2 aimed at replicating the effects found in Study 1 and examined motivation more objectively by using task choice after initial failure as a behavioral measure of motivation.

## Study 1 Method

### Design

Study 1 was an online-experiment with a one-factor between-subjects design. The between-subjects factor was feedback type with three conditions: goal attained vs. goal failed vs. no feedback (control condition). Participants all received the same high and specific goal in an intelligence test and afterward a fictitious feedback whether they attained that goal or not (or no feedback at all in the control condition; the feedback is pictured in Table 1). As dependent variables, we measured affect, self-esteem, and subsequent motivation. The same variables were assessed before the task (baseline) and after receiving the feedback.

**Table 1:** Fictitious feedback of goal-attainment/goal-failure in Study 1.

Goal-Attainment feedback:	Goal-Failure feedback:
“You have completed the intelligence-test task. Congratulations, you were able to solve at least 7 out of 10 tasks correctly. Please continue the survey.”	“You have completed the intelligence-test task. Unfortunately, you were not able to solve at least 7 out of 10 tasks correctly. Please continue the survey.”

### Participants and Procedure

We computed our required sample size with G^*^Power, optimal sample size is 111 (for between-subjects ANOVAs with three groups of Cohen’s *f*=0.3, type-I error probability *α*=0.05, and power 1-β=0.80, according to G^*^Power; Faul et al., 2007). Participants were 185 volunteers (93.5% female). Participants were randomly recruited on different online-platforms and were told that they would have the chance to test intelligence-test questions that can appear in assessment-centers. Participation was completely voluntarily; there was no payment involved. Majority of participants were employees (62.4%) of various professions (16.8% public service). Participants were not paid for participation; however, students (30.3%) received course credit if needed (only applicable to psychology students). Mean age was 28.01years (*SD*=7.0). All participants were randomly assigned to experimental conditions by using a programmed randomization filter, resulting in 67 subjects in the goal-attainment condition [34.6% female; 27.73 (*SD*=7.75) years old; 13.0% high school absolvent or higher; 17.3% employees; and 9.7% students], 53 subjects in the goal-failure condition [25.4% female; 28.58 (*SD*=6.90) years old; 11.9% high school absolvent or higher; 16.2% employees; and 7.0% students], and 65 subjects in the no-feedback control condition [33.5% female; 27.83 (*SD*=6.73) years old; 14.1% high school absolvent or higher; 17.9% employees; and 13.5% students].

After giving their consent and confirming that they are of legal age, participants answered an online-questionnaire. In this questionnaire, we assessed demographics, covariates, and baseline data for affect, self-esteem, and motivation. Participants then all received the high and specific goal to solve seven out of 10 upcoming intelligence test items. We asked how committed participants were to that goal. Participants then solved the 10 intelligence test items. After completion, participants received fictitious feedback (or no feedback in the control condition) depending on their experimental condition. Afterwards, we assessed the post-measures for affect, self-esteem, and motivation as well as perception of the goal and the feedback as manipulation checks. All study variables were assessed immediately before or after the tasks, there were no breaks in between. Finally, participants were debriefed and dismissed.

## Materials

### Intelligence Test Task

In Study 1, we used 10 intelligence test items from the freely available General Intelligence-Test by Satow (2017). These 10 items included five matrices that test spatial imagination and five number sequences that test mathematical-logical abilities. We used this task because the items all have a medium difficulty of around 0.5 (which means an item difficulty of around 50%) and participants cannot unambiguously tell if they correctly solved an item. For that reason, participants cannot be sure whether they solved the items correctly or not, which is essential for using fictitious feedback.

## Measures

### Dependent Variables

All scales that were originally in English were translated into German and then back-translated into English. Exact Cronbach’s *α* for all conditions and measurement times are listed in Table 2.

**Table 2:** Cronbach’s *α* for feedback type conditions and measurement times.

Feedback type	Time	Goal-Commitment	Affect	Self-Esteem	Motivation
Goal-Failure	1	0.88	0.89	0.81	0.80
	2	–	0.91	0.86	0.83
Goal-Attainment	1	0.80	0.89	0.79	0.70
	2	–	0.91	0.85	0.66
Control	1	0.83	0.87	0.81	0.65
	2	–	0.87	0.86	0.73

*N=185*

#### Affect

Affect was assessed with a short-scale version (Wilhelm and Schoebi, 2007) of the Multidimensional Mood State Questionnaire (MDMQ) by Steyer et al. (1997). The short-scale consists of six bipolar items (e.g., “tired – awake,” “tense – relaxed,” and Cronbach’s *α* ranged from 0.88 to 0.89) with a seven-point scale, both endpoints labeled with “*very*.”

#### Self-Esteem

State self-esteem was assessed with the subscale performance of the State-Self-Esteem Scale by Heatherton and Polivy (1991) consisting of five items. For example, one item was “I feel confident about my abilities.” Cronbach’s *α* ranged from 0.80 to 0.85. The response scale ranged from 1 (*strongly disagree*) to 5 (*strongly agree*).

#### Motivation

Motivation was assessed with three self-developed items that are based on common scales for measuring motivation. Items were “I approach even difficult tasks with motivation,” “I try everything to attain my goals,” and “When I cannot solve difficult tasks immediately, I lose interest.” Cronbach’s *α* ranged from 0.71 to 0.74. The response scale ranged from 1 (*strongly disagree*) to 5 (*strongly agree*).

### Control Variable

We measured goal-commitment as a control variable. Goal-commitment is one of the most influential moderators of the goal-performance relationship (Locke and Latham, 1990) and thus may affect the consequences of failure of a high and specific goal.

#### Goal-Commitment

Goal-commitment was assessed with three items by Hollenbeck et al. (1989) that were most appropriate for the goal-setting context. For example, one item was “I am strongly committed to this goal.” Cronbach’s *α* was 0.84. The response scale ranged from 1 (*strongly disagree*) to 7 (*strongly agree*). There were no pre-experimental differences between the groups in goal-commitment, *F*(2,182)=1.46, *p*=0.24, and *η*^2^=0.02.

### Manipulation Checks

We used several manipulation checks to make sure participants adopted the assigned high and specific goal and also to test whether the manipulation of feedback type was successful. We asked participants to repeat their assigned goal directly after the task. One hundred seventy-two participants correctly identified the assigned goal (93%). We also asked participants to rate the assigned goal on a five-point Likert scale ranging from 1 (not at all) to 5 (very). Participants perceived the assigned goal as medium to high (*M*=3.62, *SD*=0.83), difficult (*M*=3.61, *SD*=0.91), reasonable (*M*=3.46, *SD*=0.98), and fair (*M*=3.56, *SD*=0.93). We kept participants who did not correctly identify the goal in our analyses, because further ratings indicated that all participants perceived the goal as intended. Additionally, we checked whether the fictitious feedback was perceived as credible. Participants rated the feedback as credible (*M*=3.81, *SD*=1.96). There were no significant differences between the groups, *F*(2,144) =0.62, *p*=0.54, and *η*^2^=0.01 (goal-failure condition: *M*=3.66, *SD*=1.87; goal-attainment condition: *M*=3.72, *SD*=2.03).

## Study 1 Results and Discussion

### Descriptive Statistics and Intercorrelations of Study Variables

Table 3 lists the means, SDs, and correlations of all study variables. All study variables correlated in an expected manner, for example, the baseline measures correlated highly with the post-measures. In preliminary analyses, we made sure that there were no baseline differences in any of the study variables, including affect [*F*(2,182)=0.08, *p*=0.92, and *η*^2^=0.001], self-esteem [*F*(2,182)=0.02, *p*=0.98, and *η*^2^=0.00], motivation [*F*(2,182)=0.14, *p*=0.87, and *η*^2^=0.002], or goal-commitment [*F*(2,182)=1.46, *p*=0.24, and *η*^2^=0.02]. We also centered and included goal-commitment, gender, and age in our analyses. These variables did not change our results when included as covariates. We, therefore, report results of analyses without these covariates.

**Table 3:** Descriptive statistics and bivariate correlations Study 1.

S. No.		1	2	3	4	5	6	7	8	9	10	11
1.	Experimental condition	–										
2.	Performance	−0.01	–									
*Covariates*												
3.	Gender	0.12	0.06	–								
4.	Age	0.05	0.02	−0.07	–							
5.	Goal-Commitment	−0.06	−0.14	0.03	−0.13	(0.84)						
*Dependent variables*												
6.	Affect Time 1	−0.02	0.08	0.09	0.10	0.07	(0.88)					
7.	Self-Esteem Time 1	0.01	0.14	−0.01	0.16^*^	−0.01	0.44^**^	(0.80)				
8.	Motivation Time 1	0.00	0.05	0.10	0.11	0.17^*^	0.28^**^	0.52^**^	(0.71)			
9.	Affect Time 2	−0.13	0.10	0.10	0.05	0.11	0.79^**^	0.42^**^	0.24^**^	(0.89)		
10.	Self-Esteem Time 2	−0.07	0.18^*^	−0.02	0.10	−0.12	0.48^**^	0.82^**^	0.45^**^	0.55^**^	(0.85)	
11.	Motivation Time 2	−0.07	0.18^*^	0.05	0.09	0.16^*^	0.35^**^	0.47^**^	0.76^**^	0.38^**^	0.52^**^	(0.74)
Total	*M*	0.29	6.50	1.08	28.01	4.87	4.17	3.74	3.63	4.06	3.61	3.51
	*SD*	0.45	2.26	0.33	7.04	1.57	1.31	0.82	0.81	1.41	0.95	0.86
Goal-Failure	*M*	–	6.45	1.15	28.58	4.73	4.13	3.76	3.63	3.78	3.51	3.42
	*SD*	–	2.20	0.46	6.90	1.65	1.41	0.80	0.86	1.47	0.98	0.90
Goal-Attainment	*M*	–	6.40	1.04	27.73	5.13	4.15	3.73	3.59	4.46	3.69	3.59
	*SD*	–	2.23	0.21	7.51	1.39	1.31	0.81	0.80	1.40	0.92	0.80
Control	*M*	–	6.65	1.06	27.83	4.72	4.22	3.74	3.66	3.88	3.60	3.51
	*SD*	–	2.37	0.30	6.73	1.66	1.24	0.85	0.79	1.30	0.96	0.91

*N*=185; experimental condition: no failure (goal-attainment condition and control condition)=0, failure=1; and gender: female=0, other=1.

* *p<* 0.05,

** *p<* 0.01.

### Main Effects of Goal-Failure on Affect, Self-Esteem, and Motivation

We tested hypotheses using separate one-way ANCOVAs with centered baseline measures included as a covariate and with the between-subjects factor feedback type (three levels: goal attained, goal failed, and no feedback) for each dependent variable. We used *post-hoc* tests to compare the goal-failure condition with the goal-attainment condition as this comparison reflects our hypotheses.

**Figure 1:**
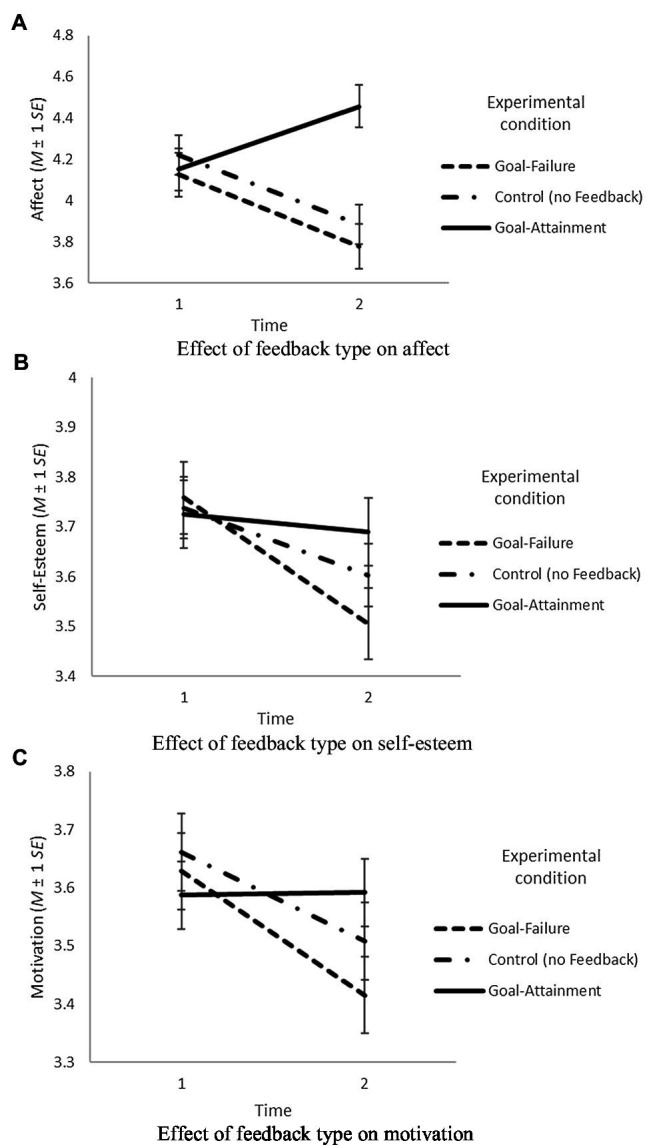
Effects of feedback type on affect **(A)**, self-esteem **(B)**, and motivation **(C)** in Study 1.

Hypothesis 1 predicted that individuals who fail the high and specific goal will show a more negative affect than individuals who attain the high and specific goal. An ANCOVA showed a significant main effect of feedback type on affect, *F*(2,181)=13.44, *p*<0.001, and *η*^2^=0.13. As planned comparisons indicated, in line with what we predicted, affect was more negative for participants who failed the goal than for participants who attained the goal. There was a statistically significant difference in affect between the goal-failure condition (*M*=3.78, *SD*=1.47) and the goal-attainment condition (*M*=4.46, *SD*=1.40) of 0.67 (*SE*=0.15), *t*(2,181)=4.47, *p*<0.001, and *d*=0.48. Thus, Hypothesis 1 was supported. The effect is depicted in Figure 1A. As illustrated, affect increased for participants who attained the goal, while it decreased for participants who failed the goal. Participants in the control condition showed a pattern similar to that of participants who failed the goal.

Hypothesis 2 assumed that individuals who fail the high and specific goal will show lower self-esteem than individuals who attain the high and specific goal. An ANCOVA showed that there was no significant main effect of feedback type on self-esteem, *F*(2,181)=2.35, *p*=0.10, and *η*^2^=0.03. However, as planned comparisons indicated, in line with what we predicted, self-esteem was lower for participants who failed the goal than for participants who attained the goal. There was a statistically significant difference in self-esteem between the goal-failure condition (*M*=3.49, *SD*=1.01) and the goal-attainment condition (*M*=3.70, *SD*=0.90) of 0.22 (*SE*=0.10), *t*(2,181)=2.16, *p*<0.05, *d*=0.22. Hypothesis 2 was supported. The effect is depicted in Figure 1B. As illustrated, self-esteem stayed at the same level for participants who attained the goal, while it was reduced for participants who failed the goal. Self-esteem levels for participants in the control conditions were in between the other two groups.

Hypothesis 3 assumed that individuals who fail the high and specific goal will show lower motivation than individuals who attain the high and specific goal. An ANCOVA showed that there was no significant main effect of feedback type on motivation, *F*(2,181)=2.32, *p*=0.10, and *η*^2^=0.03. However, planned comparisons indicated, in line with what we predicted that motivation was lower for participants who failed the assigned goal than for participants who attained the assigned goal. There was a statistically significant difference in motivation between the goal-failure condition (*M*=3.41, *SD*=1.03) and the goal-attainment condition (*M*=3.62, *SD*=0.92) of 0.21 (*SE*=0.10), *t*(2,181)=2.11, *p*<0.05, *d*=0.22. Thus, Hypothesis 3 was supported. The effect is depicted in Figure 1C. As illustrated, motivation stayed at the same level for participants who attained the goal, while it was reduced for participants who failed the goal. Motivation levels for participants in the control conditions were in between the other two groups.

In sum, all hypotheses were supported. As predicted, affect was more negative and self-esteem and motivation were reduced when the high and specific goal was failed. Interestingly, participants who received no feedback at all showed similar tendencies throughout all dependent variables as participants who failed the goal. We assume that since we chose task items with medium difficulty, participants in the no-feedback condition were not sure about their performance and assumed that they did not attain the high and specific goal; hence, they showed similar tendencies as the participants who failed the goal. We conclude that the task we used was indeed ambiguous as we intended and the uncertainty about their own performance lead to participants’ conclusion. However, to avoid uncertainty of their performance and also being dependent of the credibility of the fictitious feedback, we sought to manipulate actual performance in a second study, rather than just manipulating the feedback about the performance. Moreover, feedback in day-to-day life reflects actual performance and is not fictitious. To manipulate actual performance, we manipulated task difficulty in Study 2, so that participants can unambiguously tell how they performed and whether they attained or failed the goal. Furthermore, we sought to test the immediate behavioral effects after failure of a high and specific goal. For that reason, we used a behavioral measure of motivation in Study 2. We will describe Study 2 in detail in the following section.

## Study 2 Method

### Design

Study 2 was a laboratory experiment with a one-factor between subjects design. Between-subjects factor was goal-failure with two conditions: goal attained vs. goal failed. Participants all received a high and specific goal how many matrices they should solve in a first round. Goal-failure was manipulated through task difficulty. In a second round, participants then were asked to choose between two alternatives of the task with different difficulties. Dependent variables were motivation (task choice) in the second round as well as affect and self-esteem. Affect and self-esteem were assessed before the first round (baseline) and after the second round.

### Participants and Procedure

We computed our required sample size with G^*^Power, optimal sample size is 90 (for between-subjects ANOVAs with two groups of Cohen’s *f*=0.3, type-I error probability *α*=0.05, and power 1-β=0.80, according to G^*^Power; Faul et al., 2007). Participants were 86 volunteers (67.4% female; 61.2% employees; and 55.4% high school graduation or higher) who were recruited at several public places throughout the city at which the authors’ university is located. Participants were not paid for participation, but were able to win chocolate chips depending on their performance. Mean age was 36.70 years (*SD*=15.12). All participants were randomly assigned to experimental conditions by using a common randomization table, resulting in 41 subjects in the goal-attainment condition and 45 subjects in the goal-failure condition.

After giving their consent and confirming that they are of legal age, participants answered a first paper-pencil questionnaire. In this questionnaire, we assessed demographics, covariates, and baseline data for affect and self-esteem. Participants then all received the high and specific goal to solve four out of five matrices in the upcoming “adding-to-ten” task. Participants then tried to solve the five matrices. Participants in the goal-attainment condition received matrices that were so easy that any individual with a basic skill-level in arithmetic can solve them in the given amount of time to make sure that they all attain the assigned goal. Participants in the goal-failure condition received matrices that were so difficult that it was impossible to solve them in the given amount of time to make sure that they all fail the assigned goal. We tested whether the respective task-difficulty would lead to attaining or failing the goal in pilot studies and adjusted it accordingly. Hence, goal-failure was manipulated independently from an individual’s skill-level and solely based on our experimental manipulation of task difficulty. As intended, all participants attained or failed the assigned goal corresponding to our manipulation. After that first round, participants were asked to choose between two alternatives of the previous task with different levels of difficulty (medium vs. high, connected with different rewards). After completing that second round, we assessed the post-measures for affect and self-esteem and the manipulation check. Finally, participants received their reward of their respective amount of chocolate chips (depending on how many matrices they had solved in the second round), were debriefed and dismissed.

## Materials

### Adding-to-Ten Task

**Figure 2:**
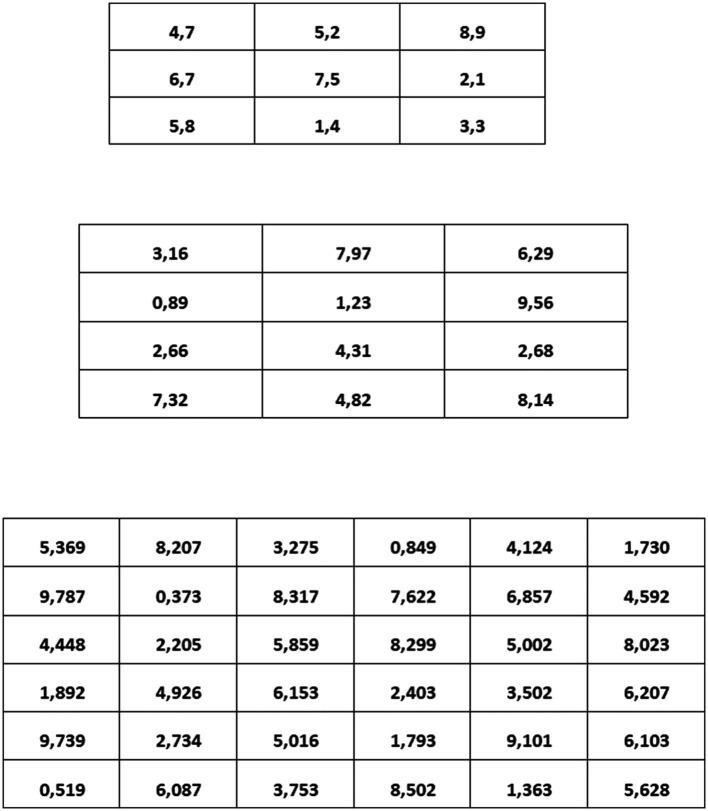
Examples for easy (top panel), medium (middle panel), and difficult (bottom panel) matrices in the “adding-to-ten” task.

In Study 2, we used the “adding-to-ten” task which has been used in several other studies (on the effects of goal-setting on unethical behavior) before (e.g., Mazar et al., 2008; Welsh and Ordóñez, 2014; Keith, 2018). The original task consists of matrices with 12 numbers with two decimal places of which two numbers sum up to 10. We used this task because it allows the respondents to unambiguously evaluate if they had solved the question correctly and because it is not viewed as one reflecting math ability (Mazar et al., 2008). In this task, participants recognize their actual performance and are not dependent on our feedback. For this study, we developed three different levels of difficulty. We varied the level of difficulty by adding more columns or more decimal places. We conducted a preliminary study to test our matrices for difficulty. The final matrices had nine numbers with one decimal place for the very easy matrices, 12 numbers with two decimal places (as in the original) for the medium difficult matrices, and 36 numbers with three decimal places for the very difficult matrices (for an example, see Figure 2). We also measured the time it took participants to solve the very easy matrices in a preliminary study. Since participants solved five very easy matrices in less than 2min, we set the high and specific goal at four out of five matrices in 2min in the goal-attainment condition. The same goal applied to the very difficult matrices in the goal-failure condition because we expected it to be impossible to solve those in 2min. In the second round, participants had another 2min to solve as many matrices as possible.

## Measures

### Dependent Variables

All Scales that were originally in English were translated into German and then back-translated into English. Exact Cronbach’s *α* for all conditions and measurement times is listed in Table 4.

**Table 4:** Cronbach’s *α* for goal-failure conditions and measurement times.

Feedback type	Time	Self-Efficacy	Risk-Taking	Perceived mental arithmetic	Affect	Self-Esteem
Goal-Failure	1	0.85	0.81	0.84	0.60	0.73
	2	–	–	–	0.72	0.77
Goal-Attainment	1	0.74	0.85	0.91	0.70	0.67
	2	–	–	–	0.75	0.60

*N=86*.

#### Motivaton (Task Choice)

Motivation was measured by task choice in the second round. Participants were asked to choose between two alternatives: To solve medium difficult matrices, receiving one chocolate for every correctly solved matrix; or to solve very difficult matrices, receiving three chocolates for every correctly solved matrix. Hence, the difficult matrices were connected with a large reward, while the medium difficult matrices were connected with a small reward. We included this payoff to have an incentive to choose the difficult matrices. Choosing the difficult matrices indicated higher motivation. Task choice was measured as choice for medium difficult matrices (0=medium difficulty) or choice for difficult matrices (1=high difficulty).

#### Affect

Affect was assessed with the same scale used in Study 1. Cronbach’s *α* ranged from 0.65 to 0.75.

#### Self-Esteem

State self-esteem was assessed with the same scale used in Study 1. Cronbach’s *α* was 0.70 at both times.

### Control Variables

We measured self-efficacy, risk-taking, and perceived mental arithmetic ability as control variables. Self-efficacy is, besides self-esteem, considered as one of the four core traits that constitute core self-evaluations (Bono and Judge, 2003). Hence, self-efficacy is expected to correlate substantially with self-esteem and as a trait may affect the consequences of goal-failure. Risk-taking was measured because it may affect which task participants choose in the second round. Mental arithmetic ability may affect how well participants perform in the “adding-to-ten” task. There were no pre-experimental differences between the groups in any of the control variables.

#### Self-Efficacy

Self-efficacy was assessed with the German version of the General Self-Efficacy Short Scale (ASKU) by Beierlein et al. (2012). The scale consists of three items, for example, “I can rely on my own abilities in difficult situations.” Cronbach’s α was 0.81. The response scale ranged from 1 (*strongly disagree*) to 5 (*strongly agree*).

#### Willingness for Risk-Taking

Willingness for risk-taking was assessed with the subscale “risk-taking” from the TCU Adolescent Social Functioning Form by the TCU Institute of Behavioral Research (2010), consisting of seven items. For example, one item was “You like taking risks.” Cronbach’s *α* was 0.84. The response scale ranged from 1 (*strongly disagree*) to 5 (*strongly agree*).

#### Perceived Mental Arithmetic

Perceived mental arithmetic ability was assessed with the subscale “attitude to fast mental arithmetic” from the “Trends in International Mathematics and Science Study” (TIMSS) student questionnaire by Wendt et al. (2017), consisting of six items. For example, one item was “Usually, I am very good at fast mental arithmetic.” Cronbach’s *α* was 0.88. The response scale ranged from 1 (*strongly disagree*) to 4 (*strongly agree*).

### Manipulation Checks

We used two manipulation checks to make sure that participants had adopted the assigned high and specific goal and that the manipulation of goal-failure was successful. We asked participants to repeat their assigned goal directly after the task. Seventy-four participants correctly identified the assigned goal (86%). We also asked participants if they attained or failed the assigned goal. All 86 participants correctly indicated that they attained the goal in the goal-attainment condition or failed the goal in the goal-failure condition. Hence, manipulation of goal-failure was successful.

## Study 2 Results and Discussion

### Descriptive Statistics and Intercorrelations of Study Variables

Table 5 lists the means, SDs, and correlations of all study variables. All study variables correlated in an expected manner. Experimental condition correlated highly with motivation (task choice), affect, and also with self-esteem. In preliminary analyses, we made sure that there were no baseline differences in any of the study variables, including affect [*t*(84)=1.72, *p*=0.09, and *d*=0.37], self-esteem [*t*(84)=0.09, *p*=0.93, and *d*=0.03], self-efficacy [*t*(84)=1.67, *p*=0.10, and *d*=0.36], risk-taking [*t*(84)=−1.12, *p*=0.27, and *d*=0.24], or perceived mental arithmetic ability [*t*(84)=−0.43, *p*=0.67, and *d*=0.09]. Some covariates seemed to correlate highly with the dependent variables, for example perceived mental arithmetic ability with motivation (task choice) or self-efficacy with self-esteem. For that reason, we centered and included these covariates in our analyses. These variables did not change our results when included as covariates. We, therefore, report results of analyses without these covariates.

**Table 5:** Descriptive statistics and bivariate correlations Study 2.

S. No.		1	2	3	4	5	6	7	8	9	10	11
1.	Experimental condition	–										
*Covariates*												
2.	Gender	0.12	–									
3.	Age	0.21	0.07	–								
4.	Self-Efficacy	−0.18	0.05	0.16	(0.81)							
5.	Risk-Taking	0.12	0.25^*^	−0.17	0.07	(0.84)						
6.	Perceived mental arithmetic	0.05	0.34^**^	0.05	0.18	0.26^*^	(0.88)					
*Dependent variables*												
7.	Affect Time 1	−0.18	0.00	−0.13	0.18	0.05	0.13	(0.65)				
8.	Self-Esteem Time 1	−0.01	0.18	0.14	0.67^**^	0.04	0.19	0.29^**^	(0.70)			
9.	Affect Time 2	−0.31^**^	0.04	−0.09	0.44^**^	0.00	0.10	0.59^**^	0.38^**^	(0.75)		
10.	Self-Esteem Time 2	−0.22^*^	0.13	0.10	0.58^**^	−0.05	0.19	0.16	0.67^**^	0.51^**^	(0.70)	
11.	Motivation (Task Choice)	−0.54^**^	0.15	−0.21	0.03	0.08	0.36^**^	0.15	−0.02	0.08	0.04	
Total	*M*	0.52	0.67	36.70	3.99	2.88	2.44	3.70	4.05	3.70	3.89	0.36
	*SD*	0.50	0.47	15.12	0.54	0.73	0.70	0.92	0.48	1.07	0.59	0.48
Goal-Failure	*M*	–	0.62	39.67	3.90	2.96	2.47	3.54	4.05	3.39	3.77	0.11
	*SD*	–	0.49	16.06	0.55	0.68	0.62	0.91	0.44	1.04	0.60	0.32
Goal-Attainment	*M*	–	0.73	33.44	4.09	2.79	2.41	3.88	4.06	4.05	4.02	0.63
	*SD*	–	0.45	13.48	0.52	0.78	0.79	0.91	0.52	1.02	0.54	0.49

*N*=86; experimental condition: no failure=0, failure=1; gender: female=0, male=1; and task choice: medium difficult=0, difficult=1.

* *p<* 0.05,

** *p<* 0.01.

### Main Effects of Goal-Failure on Affect, Self-Esteem, and Motivation (Task Choice)

We tested Hypotheses 1 and 2 using separate one-way ANCOVAs with centered baseline measure included as a covariate and with the between-subjects factor goal-failure for each dependent variable. We tested Hypothesis 3 using logistic regression with goal-failure (two levels: goal attained, goal failed) as the between-subjects factor and (motivation) task choice as dependent variable since logistic regression is recommended for dichotomous dependent variables (Mood, 2010).

**Figure 3:**
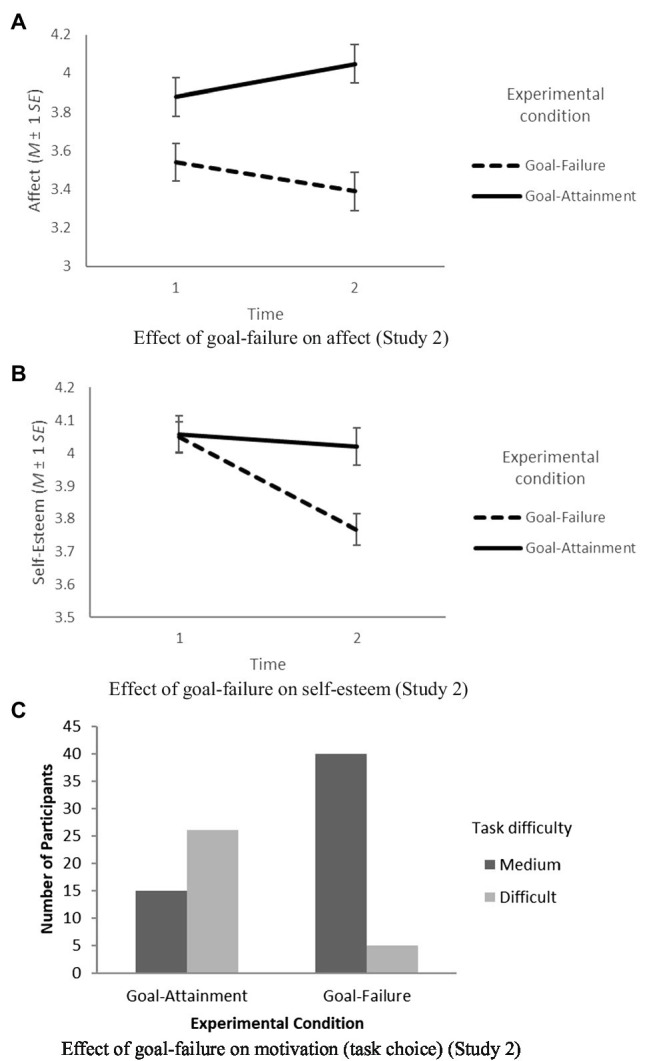
Effects of goal-failure on affect **(A)**, self-esteem **(B)**, and motivation task choice **(C)** in Study 2.

Hypothesis 1 assumed that individuals who fail their assigned high and specific goal will show a more negative affect than individuals who attain their assigned high and specific goal. An ANCOVA showed a significant main effect of goal-failure on affect, *F*(1,83)=5.64, *p*<0.05, *η*^2^=0.06, and *d*=0.37. Participants who failed their goal (*M*=3.49, *SD*=1.18) showed a significantly more negative affect than participants who attained their goal (*M*=3.94, *SD*=1.24). Hence, Hypothesis 1 was supported. The effect is depicted in Figure 3A. As illustrated, affect increased for participants who attained the goal, while it decreased for participants who failed the goal.

Hypothesis 2 assumed that individuals who fail their assigned high and specific goal will show lower self-esteem than individuals who attain their assigned high and specific goal. An ANCOVA showed a significant main effect of goal-failure on self-esteem, *F*(1,83)=7.10, *p*<0.01, *η*^2^=0.08, and *d*=0.42. Participants who failed their goal (*M*=3.77, *SD*=0.58) showed significantly lower self-esteem than participants who attained their goal (*M*=4.02, *SD*=0.61). Hence, Hypothesis 2 was supported. The effect is depicted in Figure 3B. As illustrated, self-esteem stayed at the same level for participants who attained the goal, while it decreased for participants who failed the goal.

Hypothesis 3 assumed that individuals who fail their assigned high and specific goal will show lower motivation than individuals who attain their assigned high and specific goal. We used task choice as a behavioral indicator of motivation. A logistic regression showed that goal-failure is a significant predictor of task choice, *χ*^2^(1)=27.19, *p*<0.001, OR=13.87, *d*=1.45, 95%-CI(4.5, 42.76)], with a regression coefficient of −0.26. The model explained 37.2% (Nagelkerke *R*^2^) of the variance in task choice and correctly classified 76.7% of cases. Goal-failure was associated with a decreased likelihood of choosing the more difficult task. In the goal-attainment condition, 15 participants (36.6%) chose the medium difficult task and 26 participants (63.4%) chose the highly difficult task. In the goal-failure condition, 40 participants (88.9%) chose the medium difficult task and only five participants (11.1%) chose the highly difficult task. Hence, Hypothesis 3 was supported. The results are depicted in Figure 3C.

In sum, the results of Study 2 replicate and extend the findings of our previous study. Specifically, we found support for the harmful effect of goal-failure on affect and self-esteem. As expected, after goal-failure participants showed decreased affect and self-esteem, while after goal-attainment participants showed the same or slightly higher levels of affect and self-esteem. Furthermore, we demonstrated that goal-failure affects subsequent motivation in terms of task choice. After goal-failure, the majority of participants chose the easier task and avoided the challenging task.

## General Discussion

Setting high and specific goals has long been recommended as one of the most effective motivational and leadership tools. Yet, setting high performance goals naturally leads to a considerable group of individuals who will fail that goal. Past research on goal-failure indicates that it can cause a variety of undesirable and potentially harmful effects, for the individual as well as for organizations; however, to our knowledge, there is little to no research that combines research on failure with high and specific goals that are the focus of goal-setting theory. Our research aimed at shedding light on this important topic by examining the effects of failing a high and specific goal on affect, self-esteem, and motivation; factors which may have crucial implications for organizations.

We conducted two studies to test for the expected detrimental effects of failure of a high and specific goal on affect, self-esteem, and motivation. Study 1 showed goal-failure of the assigned high and specific goal lead to a decrease in affect, self-esteem, and motivation. We replicated these effects in Study 2 and were able to show the behavioral consequences of the decreased motivation through task choice. In sum, we were able to show that the failure of a high and specific goal can trigger potentially harmful consequences for self-related factors and can hinder a person from tackling new challenges. We discuss theoretical and practical implications, limitations, and future directions of all the findings in the following sections.

### Theoretical and Practical Implications

Our findings are an important contribution to research on goal-setting theory by combining basic assumptions of achievement goal theory with goal-setting theory. Goal-setting theory focuses on those who attain the high and specific goal and states a so-called “high performance cycle” in which individuals are satisfied with their performance and enter an ever-increasing cycle of increased motivation and performance (Locke and Latham, 1990, 2002). Even though cautionary remarks have always been made about potential pitfalls when applying goal-setting (Locke and Latham, 2002), the high risk of failing that goal is widely overlooked. One theory that takes the possibility of goal-failure into account is achievement goal theory. Achievement goal theory states that goals can be framed as performance goals or learning goals. According to achievement goal theory, performance goals set an external standard. Individuals who fail that standard perceive their skills as fixed, thus, failing implies that their abilities are insufficient (Dweck and Leggett, 1988). Hence, failing of a performance goal poses a threat for the self. Individuals will perceive themselves as incapable after failing a performance goal, which can be damaging for their self-image. The high and specific goals used in goal-setting interventions are usually framed as performance goals, for example, to produce a certain amount of products, to sell a certain amount, or to enroll a certain number of customers. Combining the assumptions of both theories, one can conclude that failure of a high and specific goal has the potential to pose a threat for a person’s self. We were able to confirm this notion and found that failing a high and specific goal indeed harmed self-related factors.

Our results imply that when using goal-setting interventions in organizations, potentially harmful long-term effects should be considered. Setting high and specific goals is a very commonly used motivational tool because organizations often solely focus on the immediate results, especially on performance. However, setting high and specific goals can have serious detrimental consequences. In recent years, several undesirable effects of high and specific goals have been discussed. For example, a number of studies have explored the effects of goal-setting on unethical behavior (e.g., lying and cheating). The assumption is that individual’s attention focus is narrowed on attaining the goal, so that moral standards are ignored (Schweitzer et al., 2004; Ordóñez et al., 2009; Welsh and Ordóñez, 2014). Unethical behaviors may be particularly likely when attaining the goal is tied to monetary rewards (Jensen, 2003). Furthermore, some researchers argue that high and specific goals make destructive leadership more likely by increasing leaders’ stress to meet deadlines (Bardes and Piccolo, 2010).

Our findings show that failure of a high and specific goal can harm a person’s self and motivation. These consequences have the potential to harm not only the employee but also the organization’s results in the long-run. Our results also implicate that failure of a high and specific goal can have immediate behavioral consequences and can discourage employees from engaging in new challenges; something employees face daily in their everyday life. We recommend that organizations should find ways to sensitize supervisors and employees for the potential undesirable effects when setting high and specific goals and find ways to counteract them.

### Limitations and Future Research

A first limitation of this research is that we did not test the effects of goal-failure in an actual work-setting. Hence, we cannot be sure about the external validity of the findings. However, Study 2 was conducted in the field, at several public places with a rather heterogeneous sample of mainly working adults. Study 2 also allowed a face-to-face setting, which increases psychological realism. Given the large body of converging findings across experimental laboratory and field research on goal-setting, we assume that the used experimental designs should be suited for our investigations. Still, our experimental research should be complemented by field studies in actual work-settings, preferably by longitudinal studies that investigate long-term effects of goal-setting and goal-failure.

A second limitation of our research is that we solely used self-reports to measure the person’s affect and self-esteem. To generalize the found effects, other components of a person’s well-being should be examined, for example an individual’s physical well-being and somatic health. Research showed that the fulfillment of one’s goals plays an important role when coping with stressful events (Emmons and Kaiser, 1996). If a person is repeatedly faced with obstacles blocking the attainment of their goals, the person may be particularly susceptible to experiences of helplessness, which are associated with health risks (Brunstein et al., 1998). Future research is needed to examine the consequences of goal-failure for a person’s physical and mental health. In addition, we only measured participants’ general affect rather than discrete emotions. It is possible that discrete emotions, like anger, anxiety, or depression, provide more information on the outcomes of the goal process than generalized affect (Plemmons and Weiss, 2013). We suggest that future research examines the role of discrete emotions for processes induced by goal-failure.

Another limitation of our research is that we used a self-developed scale for measuring motivation in Study 1. Therefore, we have no information about the validity of our scale. However, we used this scale because the common validated motivation scales usually measure a more general attitude towards work, while we sought to measure motivational change toward certain work tasks. In Study 2, we included a behavioral measure of that motivational change by measuring task-choice, which is a common behavioral measure of work motivation (Thomas and Ward, 1983; Nicholls, 1984). Thereby, we were able to combine attitudinal and behavioral measures of motivation. We suggest that these findings should be complemented by a field study with an actual work task.

An additional limitation is that we were not able to examine long-term effects of failure of high and specific goals. In organizations, individuals are confronted with new goals constantly, even if they were not able to attain previous goals. Consecutive failure of high and specific goals might induce a downward spiral of harmful consequences which, in the long-run, damage the organizational outcomes, for example, reduced OCB, increased absenteeism, and disengagement from challenging tasks and burnout (Seo and Ilies, 2009; Ybema et al., 2010; Shi et al., 2013; Welsh et al., 2020). There are few studies which have investigated the effects of setting high and specific goals consecutively (Welsh and Ordóñez, 2014; Keith, 2018). For example, it was found that consecutive goal-setting can have detrimental effects on an individual’s goal-commitment and perceived fairness (Keith, 2018). In another study, consecutive performance goals increased unethical behavior by depleting self-regulatory resources (Welsh and Ordóñez, 2014). Future research should investigate the long-term consequences of failure of a high and specific goal or consecutive failure.

Furthermore, it has to be noted that our sample in Study 1 consisted mainly of female participants (93.5%). Past research found that in an experiment, after failure, male participants chose more difficult goals in a subsequent task than did female participants (Levy and Baumgardner, 1991). Additionally, it was found that individuals higher in self-esteem chose more difficult goals. We were able to control for confounding effects of base self-esteem and self-efficacy and both variables did not affect our results, which is consistent with other research on self-esteem and goal-choice (Hollenbeck and Brief, 1987). We cannot be certain that our results also apply to male individuals; however, gender did not affect our results in Study 2 and past research suggests that unambiguous feedback to insure clear failure or success on a task eliminates gender differences in future success expectancies (Feather and Simon, 1973; McMahon, 1973; Lenney, 1977). Nevertheless, future research should replicate our findings with male individuals to rule out possible gender differences.

Lastly, future research should explore methods to counteract the found undesirable effects. Drawing from achievement goal theory, one possible method may be goal-framing. While performance goals emphasize the attainment of an externally-set standard, learning goals emphasize increasing the own competence or mastering something new. When individuals fail a learning goal, they do not blame the failure on themselves, since they view their skills as changeable (Dweck and Leggett, 1988; Welsh et al., 2019). Thus, failure of a high and specific learning goal should *not* pose a threat for a person’s self and framing the goal as a learning goal may counteract the found undesirable effects.

A second possible method to counteract these undesirable effects is to let employees experience success. Past research found that success on previous tasks may breed success on subsequent tasks (Fan et al., 2020). When employees attain easy goals on previous tasks, goal-commitment increases through enactive mastery. As a result, employees increase their personally-set goals and are able to self-motivate for upcoming tasks. Hence, organizations could increase employee’s confidence and enable mastery by setting easy goals first, to create experiences of success.

A third possible method for counteracting the undesirable effects is to use self-regulatory strategies to increase one’s self-control to engage in aversive tasks. Research on the topic found that individuals who focused on the positive consequences of an aversive activity or the negative consequences for not performing it, increased their perceived self-regulatory success. Furthermore, setting goals for the activity and emotion regulation also increased self-control (Hennecke et al., 2019). Hence, when failure experiences harm an employee’s motivation and well-being, self-regulatory strategies may be used to restore those resources for subsequent tasks. Especially evocation of negative affect can increase and prolong rumination after failure experiences, which in turn can increase negative affect (Jones et al., 2013). There are various strategies that can be used to prevent detrimental effects on one’s affect. For example, an employee might use attentional deployment or focus on other aspects. After the affective state is already affected, an employee might regulate their emotions by reappraisal (Boss and Sims Jr., 2008). Thus, we recommend the use of self-regulatory and emotion regulation strategies to replenish those resources, stay persistent, and counteract effects after goal-failure.

A final strategy for counteracting undesirable effects after goal-failure might be to positively affect goal striving as well as goal revision. It has been shown that high self-efficacy and confidence in the own abilities can facilitate successful goal striving (Wolf et al., 2018). Furthermore, research found that individuals use performance-goal discrepancies to make their goal revision decisions. It was found that large discrepancies, especially over a longer period of time, led to a downward revision of their goal (Donovan and Williams, 2003). Accordingly, experiences of success lead to an upward revision of their goal. Considering the previously mentioned methods to enable mastery and to create experiences of success, we assume that these methods are also suitable to positively affect goal striving and goal revision and in turn have the potential to counteract detrimental effects after goal-failure.

## Conclusion

Our research contributes to research and practice of goal-setting by explicitly integrating research on failure with the basic recommendation of goal-setting theory and achievement goal theory. We were able to elucidate a highly possible downside of goal-setting interventions by showing that the failure of a high and specific goal can damage self-related factors like affect, self-esteem, and motivation and can also have subsequent behavioral consequences. These short-term consequences may lead to serious long-term consequences, especially when goals are failed consecutively and the person has no resources to counteract the effects. For that reason, employers need to be sensitized for the high possibility of failing a high and specific goal when using goal-setting as a motivational and leadership tool and need to take actions to counteract these undesirable effects, for example with self-regulatory or emotion regulation strategies or by experiences of success.

## Data Availability Statement

The raw data supporting the conclusions of this article will be made available by the authors, without undue reservation.

## Ethics Statement

Study 1 involving human participants was reviewed and approved by the Ethics committee of the Technical University of Darmstadt, Darmstadt, Germany. Study 2 followed the same guidelines. The participants provided their written informed consent to participate in the studies.

## Author Contributions

JH will have first authorship of this manuscript, and will also be serving as the corresponding author. The author listed in the byline has agreed to the byline order and to the submission of the manuscript in this form. All authors contributed to the article and approved the submitted version.

## Funding

This research was supported by grant from the German Research Foundation (Deutsche Forschungsgemeinschaft, grant no. KE 1377/5–1).

## Conflict of Interest

The authors declare that the research was conducted in the absence of any commercial or financial relationships that could be construed as a potential conflict of interest.

## Publisher’s Note

All claims expressed in this article are solely those of the authors and do not necessarily represent those of their affiliated organizations, or those of the publisher, the editors and the reviewers. Any product that may be evaluated in this article, or claim that may be made by its manufacturer, is not guaranteed or endorsed by the publisher.
